# Case series of catheter‐based arrhythmia ablation in 13 pregnant women

**DOI:** 10.1002/clc.24072

**Published:** 2023-07-05

**Authors:** Sára Mladoniczky, Zsófia Nagy, Csaba Földesi, Zoltán Som, Hajnalka O. Bálint, László Környei, Diána Ruzsa, Eszter Fődi, Tamás Simor, Attila Kardos

**Affiliations:** ^1^ Adult Cardiology Department Gottsegen National Cardiovascular Center Budapest Hungary; ^2^ Pediatric Cardiology Department Gottsegen National Cardiovascular Center Budapest Hungary; ^3^ University of Pécs Medical School Heart Institute Pécs Hungary

**Keywords:** ablation, arrhythmias, electrophysiology, pregnancy, zero fluoroscopy

## Abstract

**Background:**

Catheter ablation is a rarely used procedure to treat arrhythmias during pregnancy.

**Hypothesis:**

In the case of maternal arrhythmia during pregnancy, zero‐fluoroscopic catheter ablation is preferable to medical treatment.

**Methods:**

Between April 2014 and September 2021, we examined the demographic data, procedural parameters, and fetal and maternal outcomes in pregnant women undergoing ablation at the Gottsegen National Cardiovascular Center and University of Pécs Medical School, Heart Institute.

**Results:**

Fourteen procedures (14 electrophysiological studies [EPS], 13 ablations) performed on 13 pregnant women (age 30.3 ± 5.2 years, primipara *n* = 6) were studied. During EPS, 12 patients had inducible arrhythmias. Atrial tachycardia was confirmed in three, atrioventricular re‐entry tachycardia via manifest accessory pathway (AP) in three, and via concealed AP in one case. Atrioventricular nodal re‐entry tachycardia was confirmed in three and sustained monomorphic ventricular tachycardia in two cases. Eleven radiofrequency ablation (84.6%) and two cryoablation (15.4%) were performed. The electroanatomical mapping system was used in all cases. Transseptal puncture was performed in two cases (15.4%) due to left lateral APs. The mean procedure time was 76.0±33.0 minutes. All procedures were performed without fluoroscopy. No complications occurred. During the follow‐up, arrhythmia‐free survival was achieved in all cases, but in two patients, we used antiarrhythmic drugs (AADs) to achieve it. APGAR score was within the normal range in all cases [median (interquartile range), 9.0/10.0 (9.0–10.0/9.3–10.0)].

**Conclusions:**

Zero‐fluoroscopic catheter ablation was an effective and safe treatment option for our 13 pregnant patients. Catheter ablation may have less side effects on fetal development than the use of AADs during pregnancy.

## Graphical abstract

We presented a case series of 13 pregnant women who underwent zero‐fluoroscopy catheter ablation due to arrhythmias. We examined the demographic and procedural data and fetal and maternal outcomes. Zero‐fluoroscopic catheter ablation was effective and safe and may have less side effects on fetal development than the use of antiarrhythmic drugs.

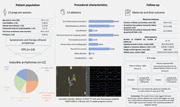

## 1 INTRODUCTION

Symptomatic arrhythmias during pregnancy require a complex electrophysiological approach. Out of 100 000 pregnant women, 67 are known to have arrhythmias. Supraventricular tachycardia (SVT), such as atrioventricular nodal re‐entry tachycardia (AVNRT) and atrioventricular re‐entry tachycardia (AVRT), is the most common arrhythmia causing clinical symptoms. However, atrial fibrillation (AF), atrial flutter, and ventricular tachycardia (VT) can also manifest during pregnancy. Persistent arrhythmias or rapid ventricular rate response may cause maternal and fetal hemodynamic deterioration.

Many patients with symptomatic arrhythmias have been successfully treated with medical therapy. However, antiarrhythmic drugs (AADs) should be used with caution because of the adverse effects on maternal and fetal life.

Fetal risks may include preterm birth, intrauterine growth retardation, small for gestational age (SGA), respiratory distress syndrome, and congenital heart disease, which occurred in 20% of pregnancies treated with specific AADs such as beta‐blockers (BB), propafenone, verapamil, or amiodarone.^1^, ^2^, ^3^

Although catheter ablation is a treatment of choice for SVT (recommendation IB), it is only recommended in pregnant patients with drug‐refractory and poorly tolerated SVT (IIa C) according to the current guidelines.^4^, ^5^

Catheter ablation is not widely used in the treatment of pregnant patients because of the potential risks of the invasive procedure (anesthetic agents, thromboembolic complications, difficulty in accessing the vessel) and radiation exposure. During ablation, radiation exposure has been minimized by the development of nonfluoroscopic catheter navigation techniques. Nowadays, physicians should consider zero‐fluoroscopic catheter ablation as a less risky treatment than the use of harmful AADs in this specific patient group.^1^, ^2^

The current study aimed to report on our experience to demonstrate the safety and efficacy of catheter ablation in cardiac arrhythmias during pregnancy.

## 2 METHODS

### 2.1 Study design and patient population

We conducted a double‐center retrospective case series study. We examined the population of patients undergoing catheter ablation for cardiac arrhythmias during pregnancy from April 2014 to September 2021 at the Gottsegen National Cardiovascular Center (GOKVI) and the University of Pécs (Faculty of Medicine). This study complies with the Declaration of Helsinki and was approved by the National Ethics Committee.

Our patient population included 13 pregnant women who underwent 14 electrophysiological studies (EPS) and 13 catheter ablations due to highly symptomatic or therapy‐refractory cardiac arrhythmias. In one patient, only EPS was performed, and, in another case, redo ablation was required. Arrhythmias included any episodes of atrial tachycardia (AT), AF, AVRT, AVNRT, premature ventricular contractions (PVC), and VT during pregnancy. Patients' symptoms were most often palpitations and less frequently chest complaints. One patient did not have palpitations, the first symptom was the appearance of heart failure (dyspnea, lower limb edema). None of the patients had syncope.

Cardiac examination, including medical history, physical examination, 12‐lead electrocardiogram (ECG), transthoracic echocardiography, 24‐hour Holter recording, and cardiac magnetic resonance imaging in case of VT was recorded in these patients, preprocedurally.

### 2.2 Preprocedural management and indications for catheter ablation

All patients had symptomatic tachyarrhythmias. We opted for EPS and catheter ablation because arrhythmias may negatively impact hemodynamics and, therefore, fetal development. Some arrhythmias required elective intervention and some required emergent management. Patients with severe symptoms who were admitted as emergencies required a single or multiple low‐dose antiarrhythmic treatments for rate control or heart failure symptoms. In these emergency cases, telemetry monitoring was also required before the intervention. Patients admitted electively generally did not require medication before the procedure.

We chose catheter ablation because all patients included in the study had severe arrhythmia‐related symptoms or drug‐refractory arrhythmias. The electrophysiologists decided on the treatment plan together with the gynecologist and the cardiologist who had previously treated the patient. Patients were informed about the potential risks of procedure failure, radiation exposure, and complications before the catheter ablation. All patients have given written consent for the ablation procedure and the use of X‐rays if necessary.

### 2.3 EPS and catheter ablation

The EPS and ablation procedure was performed in conscious sedation. If possible, AADs were discontinued at least 2 weeks before the procedure. The access sites were the right femoral vein and the right internal jugular vein. We used the CARTO electroanatomical mapping (EAM) system or the NavX‐EnSite EAM system for mapping to achieve zero‐fluoroscopy procedures. Electroanatomical reconstruction of the right atrium, including critical anatomical landmarks such as the right ventricle outflow tract (RVOT), the tricuspid annulus, the His bundle region, and the coronary sinus ostium, was performed. Standard EPS was required, then other diagnostic catheters were used, such as decapolar coronary sinus catheter, quadripolar right ventricular catheter, and His catheter. In some cases, intravenous isoproterenol was used to induce arrhythmias. The anticoagulation strategy during the procedure involved the administration of intravenous bolus unfractionated heparin (usually 5000 U). Transseptal puncture was performed during two procedures because of the diagnosis of the left lateral accessory pathway (AP). The transseptal puncture was performed under intracardiac echocardiography (ICE) guidance with zero fluoroscopy (Supporting Information: Figure 1]. ICE catheter was placed in the right atrial free wall to visualize the superior vena cava and guide the advancement of the long sheath (SL0; St. Jude Medical) and Brockenbrough needle (BRK; St. Jude Medical). The needle and the long sheath were rotated clockwise, slowly retracted, and finally dropped into the fossa ovalis, and then the transseptal puncture was performed. Finally, echocardiographic contrast injection through the needle confirmed that the needle tip was correctly introduced into the left atrium (LA). Then, ICE was used to check the contact between the ablation catheter and the tissue and to control pericardial fluid at the end of the procedure.^2^

### 2.4 Follow‐up

Our study included the follow‐up of the patients, analysis of arrhythmia‐free survival, and complete details of pregnancy, delivery, and child development. Both obstetric and neonatal outcomes were determined, including the circumstances of delivery (number of weeks of gestation, cesarean section (C‐section), or per vias naturales), complications during delivery, and the birth weight, length, and APGAR score of the newborns. Birth weight was analyzed separately for those who had undergone clinically successful catheter ablation and were not taking AADs and those who had to take AADs for some reason. The two groups were compared using the Mann–Whitney *U* test.

The length of the follow‐up depended on the time of intervention, the average follow‐up period was 30.0 ± 25.0 months. We aimed to perform an outpatient visit 3 and 12 months after ablation, including a physical examination, 12‐lead ECG, and Holter or transtelephonic ECG monitoring.

### 2.5 Statistical analysis

In statistical analysis, data were expressed as frequencies and percentages. Continuous variables were expressed as mean ± standard deviations. A Mann–Whitney *U* test was used to compare groups. Statistical analysis was performed using IBM® SPSS® Statistics version 22. A *p* value less than .05 was considered significant.

## 3 RESULTS

We collected data on characteristics of pregnant women, such as parity and gestational age at the time of the procedure, comorbidities, and valvular heart disease. We analyzed the reason for ablation, the arrhythmia diagnosed during the EPS, the used ablation technique, energy source, and EAMs, and gathered other procedural parameters and periprocedural complications. The recurrence of arrhythmias, the clinical success rate, and the need for antiarrhythmic medication was analyzed during the follow‐up period. We analyzed fetal outcomes, including the gestational week at birth, mode of delivery (C‐section or vaginal), birth weight, and APGAR scores.

### 3.1 Patient characteristics (Table 1)

We studied 13 pregnant women who underwent EPS and/or catheter ablations. Table 1 shows the baseline demographic data. The mean age of the patients was 30.3 ± 5.2 years. Five women (38.5%) had comorbidities, including hypertension, hypothyroidism, Leiden mutation or deep vein thrombosis, gestational diabetes mellitus, and depression. Two patients (15.4%) had valvular heart disease; one patient underwent mitral valve repair surgery for papillary muscle rupture and infective endocarditis at the age of 23 years for mitral valve prolapse. Another patient with valvular heart disease had mitral valve prolapse with mild‐to‐moderate mitral valve regurgitation. One patient (7.7%) had heart failure with reduced ejection fraction (HFrEF), which developed at the base of tachycardia‐induced cardiomyopathy. In six patients (46.2%), preoperative medications, including BB, verapamil, ivabradine, and propafenone, were used. Two patients were on ivabradine therapy before ablation, the indications were AVRT via manifest AP in one case and RVOT PVC in the other. In the first case, drug therapy could be discontinued after successful ablation. In the second case, after ablation, verapamil therapy was started as a first step, but was changed to bisoprolol and dihydralazine therapy due to heart failure symptoms. In three patients, only BB was given before ablation with the indication of AVRT (via concealed AP), AVNRT, and right atrial re‐entry AT. In all three patients, BB therapy could be stopped after successful ablation. The sixth patient, who remained on AADs before ablation, underwent two ablation procedures for right atrial focal AT. Before the first ablation procedure, she received bisoprolol and ivabradine therapy. After the first failed ablation, her medication was changed to metoprolol and propafenone. She remained on this therapy after the second ablation and is currently taking it to achieve freedom from arrhythmias. The time since the first arrhythmia diagnosis ranged widely, at the mean of 44.0 ± 53.0 months. Some patients had tachyarrhythmia episodes since childhood, while others had suffered from it for only weeks. Six women (46.2%) had their first, five women (38.5%) had their second, and two women (15.4%) had their third pregnancy at the time of the procedure. Gestational age was 24.0 (20.0‐27.0) weeks on average at the time of the ablation procedure.

**Table 1:** Baseline demographic data.

Number of patients (*n*)	13
Maternal age (years) ± mean (SD)	30.3 ± 5.2
Comorbidity, *n* (%)	5 (38.5)
Valvular heart disease, *n* (%)	2 (15.4)
HFrEF (tachycardia‐induced cardiomyopathy), *n* (%)	1 (7.7)
Patients on preoperative medication, *n* (%)	6 (46.2)
Duration of arrhythmia (months)	44.0 ± 53.0
Parity	
First pregnancy, *n* (%)	6 (46.2)
Second pregnancy, *n* (%)	5 (38.5)
Third pregnancy, *n* (%)	2 (15.4)
Gestational week at the procedure (week), median (IQR)	24.0 (20.0–27.0)

Abbreviation: HFrEF, heart failure with reduced ejection fraction.

### 3.2 Arrhythmia characteristics (Table 2)

All women gave their informed consent for ablation and accepted the use of X‐rays if necessary. Arrhythmia characteristics are shown in Table 2. A total of 14 interventions were performed on 13 pregnant patients. There was one pregnant woman who underwent two ablation procedures. A total of 14 EPS and 13 ablations were achieved. In one case, only EPS was performed, and AVRT via manifest septal AP was found. In this patient, we diagnosed a low‐risk septal AP according to the anterograde effective refractory period of the AP (370 milliseconds), so radiofrequency ablation (RFA) was unnecessary. Of the 12 patients with inducible tachycardia requiring intervention, three patients (25.0%) had right AT. Of these three patients with right AT, two patients (16.6%) had focal AT, and one patient (8.3%) had AT with a re‐entry mechanism. AVRT was found in four patients (33.3%), three of them had AVRT via manifest AP (25.0%), consistent with Wolff–Parkinson–White syndrome, and one patient had a concealed AP (8.3%). The localization of the AP was left lateral in two cases, mid‐septal in one case, and posteroseptal in one case. Supporting Information: Figure 2 shows successful catheter ablation of the left lateral AP with zero‐fluoroscopy using the CARTO EAM. AVNRT was found in three cases (25.0%); two of them (16.6%) were diagnosed with typical “slow–fast” AVNRT, and one (8.3%) with atypical “slow–slow” AVNRT. Sustained monomorphic VT (smVT) was diagnosed in two patients (16.6%).

**Table 2:** Arrhythmia characteristics.

Number of procedures, *n*	14
Number of only EPS, *n*	1
Number of EPS + ablation, *n*	13
Patients with inducible tachycardia, *n*	12
AT, right atrial, *n* (%)	3 (25.0)
AT focal, *n* (%)	2 (16.6)
AT re‐entry, *n* (%)	1 (8.3)
AVRT, *n* (%)	4 (33.3)
AVRT via manifest AP (WPW syndrome), *n* (%)	3 (25.0)
AVRT via concealed AP, *n* (%)	1 (8.3)
AVNRT, *n* (%)	3 (25.0)
Typical “slow–fast” AVNRT	2 (16.6)
Atypical “slow–slow” AVNRT	1 (8.3)
smVT, *n* (%)	2 (16.6)

Abbreviations: AP, accessory pathway; AT, atrial tachycardia; AVRT, atrioventricular re‐entry tachycardia; EPS, electrophysiological studies; smVT, sustained monomorphic ventricular tachycardia.

### 3.3 Procedural characteristics (Table 3)

Procedural characteristics are shown in Table 3. In the 13 cases where induced tachycardia required intervention, ablation was performed. RFA was performed in 11 cases (84.6%) and cryoablation in two cases (15.4%). All the ablations (100.0%) were performed using an EAM system. In 11 cases (84.6%), we used the CARTO system for three‐dimensional mapping. The NavX‐EnSite precision mapping system was used in two cases (15.4%). All the ablation procedures (14 out of 14) were zero‐fluoroscopy procedures. Transseptal puncture was performed in two cases (14.3%), where left lateral AP was found during the EPS. ICE was used in 5 of the 13 cases (38.5%). The mean duration of the procedures was 76.0 ± 34.0 minutes. There were no complications observed during the procedures.

**Table 3:** Procedural characteristics.

Number of EPS + ablation, *n*	13
Ablation technique, energy source	
RF ablation, *n* (%)	11/13 (84.6)
Cryoablation, *n* (%)	2/13 (15.4)
Electroanatomical mapping system, *n* (%)	13/13 (100.0)
CARTO, *n* (%)	11 (84.6)
NavX‐EnSite, *n* (%)	2 (15.4)
Zero‐fluoroscopy ablation, *n* (%)	13/13 (100.0)
Transseptal puncture, *n* (%)	2/13 (15.4)
ICE, *n* (%)	5/13 (38.5)
Procedure time (min), mean (SD)	76.0 ± 34.0
Complications	0

Abbreviations: EPS, electrophysiological studies; ICE, intracardiac echocardiography; RF, radiofrequency.

### 3.4 Short‐ and long‐term follow‐up data

The clinical outcome depended on the procedural success, the recurrence of arrhythmias, the need for medical treatment, long‐term follow‐up data, and complete data on pregnancy, delivery, neonatal birth characteristics such as birth weight and APGAR score, and child development.

#### 3.4.1 Procedural success and medical treatment (Table 4)

The acute procedural success was defined as no return of arrhythmia during the 30‐minute waiting period after the procedure, and no arrhythmia could be induced by pacing or medication during the procedure. The acute procedural success was 11 out of 13 procedures (84.6%). In two patients, we performed unsuccessful ablations. One patient had a second ablation procedure because of recurrent right focal AT. No arrhythmia recurrence was observed after the redo ablation procedure with AADs (metoprolol and propafenone therapy), and no complications were observed during pregnancy. In the other unsuccessful case, a 20‐week pregnant patient underwent an ablation procedure for smVT, but VT was inducible after ablation. In this patient, the frequency and duration of VT were significantly reduced with verapamil and bisoprolol at first‐line, but later she remained on verapamil therapy alone during follow‐up. However, pregnancy and delivery were complication‐free with these AADs.

In addition to these two patients who remained on AADs (BB/propafenone/verapamil/ivabradine) due to an unsuccessful ablation procedure, one patient required medication for HFrEF (EF: 35%). In this case, ablation was performed for RVOT smVT (Supporting Information: Figure 3.). After successful RVOT VT RFA, EF improved (EF: 50%) after a few days. We decided to continue bisoprolol therapy, but the indication was heart failure with recovered left ventricular EF. In addition, the patient received dihydralazine therapy for the treatment of heart failure. In summary, 3 of the 13 patients (23.1%) required AADs during pregnancy.

In this group of patients who remained on AADs (*n* = 3), two of the three patients (66.7%) had their first pregnancy at the time of the intervention. They had a wide range in age (19, 30, and 39 years; mean 29.3 ± 6.9 years). All of them had onset of symptoms in adulthood (between 1 and 24 months before the time of intervention, mean 12.0 ± 8.0 months).

#### 3.4.2 Long‐term follow‐up

The average long‐term follow‐up time was 30.0 ± 25.0 months, depending on the date of the intervention. In all cases, we planned an outpatient visit of 3 and 12 months after ablation for our patients. Seven of the 13 patients (53.9%) did not attend an outpatient visit within 1 year of catheter ablation, despite their planned appointment. Based on the telephone interview during data collection, they were asymptomatic and therefore did not need a follow‐up visit. There was no recurrence of arrhythmia in any of the pregnant women who underwent catheter ablation. However, two pregnant women (15.4%) required AAD treatment to achieve long‐term freedom from arrhythmias. Routine Holter monitoring was performed in four patients (30.8%) without symptoms and no arrhythmia was found in any patient. In one case, routine transtelephonic ECG was achieved, which did not confirm any arrhythmias.

Table 4 shows the patients' acute procedural success and long‐term follow‐up data.

**Table 4:** Procedural success and long‐term follow‐up data.

Procedural success, *n* (%)	11/13 (84.6)
Patients on medical treatment after ablation, *n* (%)	3 (23.1)
Duration of follow‐up (months), mean (SD)	30.0 ± 25.0
Arrhythmia‐free patients during the follow‐up period, *n* (%)	13/13 (100)
Arrhythmia‐free patients during the follow‐up period off AAD, *n* (%)	11/13 (84.6)

Abbreviation: AAD, antiarrhythmic drug.

**Table 5:** Fetal outcome.

Number of childbirth (*n*), number of infants (*n*)	13, 13
Gestational week at birth, median (IQR)	38.0 (36.0–39.0)
Vaginal or Cesarean delivery, *n* (%)/*n* (%)	4 (30.8)/9 (69.2)
Reason for C‐section	
Fetal/maternal, *n* (%)/*n* (%)	5 (55.6)/4 (44.4)
Weight at birth (g), mean (SD)	2989 ± 834
APGAR scores, median (IQR)	9.0/10.0 (9.0–10.0/9.3–10.0)
Gender of the newborns (girl/boy), *n* (%)/*n* (%)	3 (23.1)/10 (76.9)

Abbreviations: IQR, interquartile range.

#### 3.4.3 Fetal outcome (Table 5)

The fetal outcome included the gestational week at birth, mode of delivery, birth weight, and APGAR score in this patient population.

At a median of 38.0 (interquartile range [IQR]: 36.0–39.0) weeks of gestation, 13 healthy newborns were delivered. Vaginal delivery was performed in four cases (30.8%). C‐section was performed in nine cases (69.2%), mostly (*n* = 5, 55.6%) for fetal reasons (cephalopelvic disproportion, SGA, umbilical flowmetry detected reverse flow signals, fetal bradycardia). In four cases (44.4%), a gynecologist decided to perform a C‐section for maternal reasons (maternal tachyarrhythmia, uterine myoma, older maternal age, previous C‐section, previous cardiac surgery). No cardiac‐related adverse event occurred during delivery. The average birth weight was 2989 ± 834 g, within the normal range. APGAR values were all within the normal range [median (IQR) 9.0/10 (9.0–10.0/9.3–10.0)]. Three of the 13 newborns were girls (23.1%), and 10 were boys (76.9%). All babies developed well during the long‐term follow‐up.

Finally, birth weight was analyzed separately for those who underwent clinically successful catheter ablation and were not taking AADs (Group 1) and those who had to take AADs for some reason (unsuccessful procedure, *n* = 2; reduced ejection fraction, *n* = 1) (Group 2). There were 10 babies in Group 1, with an average birth weight of 3253 ± 458 g. There were three babies in Group 2. The first was the baby of the patient who had undergone two interventions without permanent success and was born with a birth weight of 1990 g at 34 weeks' gestation. In the second patient, despite unsuccessful VT RFA, the baby weighed 3480 g and was born at 39 weeks' gestation. In the last case presented, where reduced EF required AAD therapy, the fetus was SGA and was born by planned C‐section at 860 g at 30 weeks' gestation. In Group 2, the average birth weight was 2110 ± 1314 g; the weighted average birth weight per week of birth in this group was 2225 g. The difference in average birth weight between the two groups, probably due to the small number of cases, did not show statistical significance (*p*  > .05). However, to indicate the trend, it may be sufficient to show the average birth weights of the two groups. The difference between APGAR score and gestational age also showed no statistically significant difference (*p* > .05).

## 4 DISCUSSION

Our study has shown that SVT is the most common arrhythmia during pregnancy. Pregnant patients with persistent severe symptomatic arrhythmias require rhythm control therapy. Zero‐fluoroscopic catheter ablation can offer a safe and effective way to treat pregnant women with sustained symptomatic arrhythmias.

Although patients with persistent maternal arrhythmias are relatively rare,^1^ Vaidya et al.^3^ found that the prevalence of arrhythmias during pregnancy increased between 2000 and 2012. AF and SVT were the most common arrhythmias during pregnancy, with VT being relatively rare.^2^, ^3^ Similar findings were found in our study population: AVRT was the most frequent arrhythmia, and ventricular arrhythmias were significantly less frequent than SVT. Even though most arrhythmias during pregnancy are benign, Ramlakhan et al.^6^ found that any arrhythmia can increase the risk of maternal and fetal adverse events or even cause high maternal mortality.

AAD therapy may also be harmful during pregnancy. Although the ESC guideline still states that this is the treatment of choice for maternal arrhythmias.^4^ Procainamide, adenosine, digoxin, CCB, and BB are considered safe to use during pregnancy (IC recommendation), although they may have undesirable side effects (e.g., low birth weight).^6^, ^7^, ^8^ Williams et al.^8^ examined in detail the effects of each group of AADs on the fetus. They found that BBs are known to reduce birth weight. CCB can cause hypotension and tocolysis, but to our knowledge has no effect on birth weight. BB, propafenone, and CCB were used in our study population only if necessary. The reasons for the need to use drug therapy were undetermined arrhythmia or HFrEF. In these cases, birth weight showed a lower trend than in those who had a successful ablation and did not take AADs during pregnancy, although the difference was not significant.

In pregnant patients, catheter ablation is recommended only in drug‐refractory SVT (IIa C).^4^ Koźluk et al.^2^ found that only 0.2% of ablation procedures are performed in pregnant women. Therefore, there are few articles in the literature on this topic, and most of them are case reports of catheter ablation during pregnancy.^9^, ^10^, ^11^, ^12^ To the best of our knowledge, we have studied one of the largest patient populations published in the literature.^2^, ^13^

The low number of ablation procedures performed during pregnancy is probably due to the fear of using X‐rays. There are two main biological effects of radiation: tissue reactions (deterministic effects) and stochastic effects, of which carcinogenicity is the most worrying.^14^ There are several additional problems with X‐rays during pregnancy, such as ethical issues, although radiation doses during catheter ablation can be limited to safe levels. According to Mattsson et al.,^15^ short‐term determinative effects can take the form of prenatal necrosis at implantation and cell death during organogenesis—which can cause growth retardation, developmental abnormalities, and neurogenetic disorders such as microcephaly, and intellectual or developmental disabilities. These effects are expected with high dose exposures (500 mGy or more) and are not expected with lower absorbed radiation (100 mGy or less). Deterministic effects also depend on gestational age, so although this is not clearly stated in the recommendation, Driver et al.'s^1^ practical experience suggests that catheter ablation should be delayed until the second trimester. However, this may present a technical challenge for the surgeon in the third trimester. In addition to short‐term effects, long‐term stochastic effects occur not only in the pregnant patient but also in the fetus. For the fetus, the risk of developing cancer later in life is also increased if the absorbed radiation dose is below 500 mGy, but it is much less expected if it is below 100 mGy.^15^ The average effective dose is 3.2 mSv during EPSs and 15.2 mSv during catheter ablation, although this can be reduced to zero using EAMs.^14^ In a recently published meta‐analysis, Kanitsoraphan et al.^16^ found that zero‐fluoroscopic catheter ablation of arrhythmias is as effective and safe as conventional catheter ablation.

In our patient population, catheter ablation was a reliable, effective, and safe choice for pregnant patients with zero fluoroscopy. Williams et al.^8^ claims that catheter ablation is performed safely during pregnancy. However, it should be performed in carefully selected patients with clinically significant drug‐refractory tachyarrhythmias with adverse effects on the mother and fetus.^13^ Therefore, in our experience, physicians should consider catheter ablation as first‐line therapy before AADs in a carefully selected pregnant patient population. However, the risk of intervention should be reduced, and pregnant women with arrhythmias should only be treated in high‐volume experienced centers, according to the recent ESC guidelines.^4^ An experienced operator using an EAM can successfully ablate and eliminate arrhythmias in pregnant patients with zero fluoroscopy. However, the intervention should preferably be postponed to the second trimester, as mentioned above. We have had no perioperative complications or adverse maternal/fetal events during delivery. Our study has done a long‐term follow‐up, and none of the children suffer from any developmental abnormality. There are few publications available on this topic in the literature. The larger studies on ablation treatment of arrhythmias in pregnancy have only examined maternal outcomes.^17^ Williams et al.^8^ investigated the effects of AAD on the fetus and birth weight but did not examine the long‐term development of the children.

Our case series study suggests that catheter ablation may be a safe and effective therapeutic option in a broader, well‐selected patient population of pregnant women, and more studies with large number of cases are needed in the future to confirm appropriate patient selection for ablation in this specific population.

## 5 CONCLUSIONS

Persistent maternal tachyarrhythmia is not common but is an essential topic in clinical practice, as the prevalence of maternal arrhythmias increased between 2000 and 2012.^3^ Untreated maternal arrhythmias can adversely affect fetal development or, if treated with AAD, cause lower birth weight and other complications.^6^, ^8^, ^17^ Catheter ablation is considered safe in pregnant patients in a carefully selected patient population,^8^, ^13^ but current guidelines recommend it only for symptomatic and drug‐refractory maternal arrhythmias.^5^ The results of our study suggest that zero‐fluoroscopic ablation has several advantages over long‐term antiarrhythmic drug therapy and can be performed safely with a high success rate among a well‐selected population of pregnant women. However, long‐term clinical outcomes and safety need to be investigated in larger cohorts, since most of the literature on this topic is based on case reports.^9^, ^10^, ^11^, ^12^

### 5.1 LIMITATIONS

Our study is a case series of 13 pregnant patients, which is a too small study population to provide statistically relevant data about the safety and efficacy of catheter ablation procedures in pregnancy. Further studies in a larger number of patients are needed to establish and support with statistically relevant data that zero‐fluoroscopic catheter ablation can be a first‐line therapy versus antiarrhythmic drugs in selected pregnant patients.

## CONFLICT OF INTEREST STATEMENT

The authors declare no conflict of interest.

## Supporting information

Supporting information.

## Data Availability

The data that support the findings of this study are available on request from the corresponding author. The data are not publicly available due to privacy or ethical restrictions.
