# Supplementary material for: Case series of catheter‐based arrhythmia ablation in 13 pregnant women
Source: Clin Cardiol. 2023 Jul 5;46(8):942–9. doi: 10.1002/clc.24072 (PMC10436797; doi:10.1002/clc.24072)
Supplement: Supplementary file 1 — Supporting information. [file CLC-46-942-s001.docx]

1. SUPPLEMENTARY FIGURES


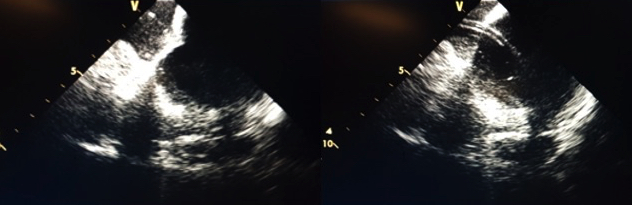


Supplementary Figure 1. Images of an ICE-guided transseptal puncture

Once the origin of arrhythmia has been identified, radiofrequency applications or, in some cases, cryoapplications were performed according to standard techniques. A successful ablation procedure was identified as the termination of the arrhythmia or the substrate, and no induction with stimulation or even intravenous isoproterenol occurred during the 30-minute waiting period. Perioperative complications were assessed in all cases. After the procedure, all patients had repeated 12-lead ECGs and were observed for at least one day.


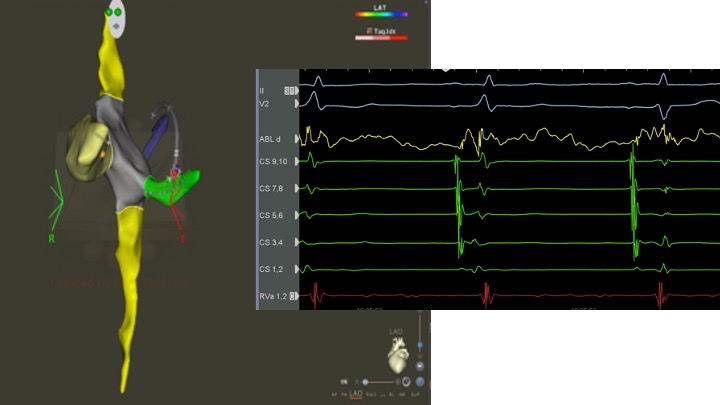


Supplementary Figure 2. Successful catheter ablation of the left lateral AP with zero fluoroscopy using the CARTO EAM

Our case is a 39-year-old patient, 29 weeks pregnant, with palpitations since childhood, but her symptoms worsened during the second pregnancy, so we performed EPS. Through right femoral venous access, we mapped the anatomy of the RA, RV, and sinus coronarius with a Navistar Thermocool STD catheter using CARTO EAM. Left AP was found with retrograde eccentric decremental conduction (RAPERP 500/200 msec) with an anterograde AP ERP of 400 msec. AVRT was inducible with a cycle length of 400 msec. After ICE-guided transseptal puncture, RF ablation was used to destroy the pathway conduction in the left lateral position. Tachycardia was not inducible after a 30 min waiting time.


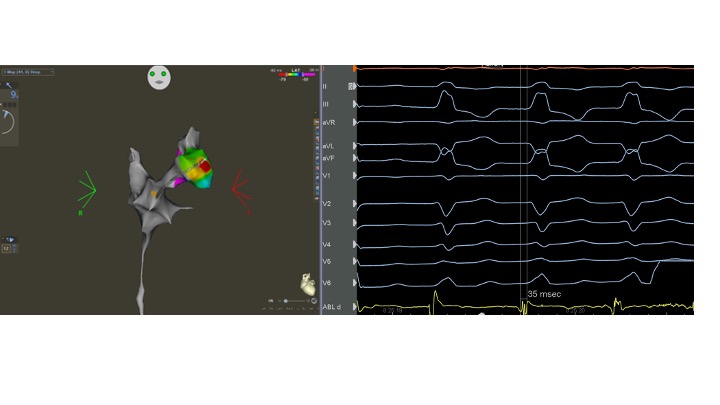


Supplementary Figure 3. Successful catheter ablation of RVOT VT with zero fluoroscopy using the CARTO EAM

Our case is a 40-year-old patient, 27 weeks pregnant. During routine 12 lead ECG examination, frequent PVC was found. During the Holter scan, non-sustained VT (nsVT) was observed, and BB therapy was started. IUGR was found during gynecological follow-up. She was then referred to our center for implantable cardioverter defibrillator (ICD) implantation. She had no arrhythmia-related symptoms, but frequent nsVT and smVT were found during telemetric monitoring. Echocardiographic examination showed reduced ejection fraction. Cardiac MR imaging confirmed the diagnosis of reduced left ventricular ejection fraction (LVEF 35%). Arrhythmogenic right ventricular dysplasia was excluded, and dilated cardiomyopathy was diagnosed. We decided to perform catheter ablation. Through right femoral venous access using CARTO EAM with a Navistar Thermocool STD catheter, we mapped the anatomy of the RVOT region. Based on the activation map (earliest signal was 35 msec before the QRS) and pace map (96% match), we applied radiofrequency applications of 20-30-40 W in the anterior septal region of the RVOT. At the end of the procedure, PVC was eliminated and VT was not inducible.
